# Melanoma and Nanotechnology-Based Treatment

**DOI:** 10.3389/fonc.2022.858185

**Published:** 2022-03-09

**Authors:** Hong Zeng, Jia Li, Kai Hou, Yiping Wu, Hongbo Chen, Zeng Ning

**Affiliations:** Department of Plastic and Cosmetic Surgery, Tongji Hospital, Tongji Medical College, Huazhong University of Science and Technology, Wuhan, China

**Keywords:** melanoma, nanotechnology, drug delivery, cytotoxicity, metastasis

## Abstract

Melanoma is a malignant tumor arising in melanocytes from the basal layer of the epidermis and is the fifth most commonly diagnosed cancer in the United States. Melanoma is aggressive and easily metastasizes, and the survival rate is low. Nanotechnology-based diagnosis and treatment of melanoma have attracted increasing attention. Importantly, nano drug delivery systems have the advantages of increasing drug solubility, enhancing drug stability, prolonging half-life, optimizing bioavailability, targeting tumors, and minimizing side effects; thus, these systems can facilitate tumor cytotoxicity to achieve effective treatment of melanoma. In this review, we discuss current nanosystems used in the diagnosis and treatment of melanoma, including lipid systems, inorganic nanoparticles, polymeric systems, and natural nanosystems. The excellent characteristics of novel and effective drug delivery systems provide a basis for the broad applications of these systems in the diagnosis and treatment of melanoma, particularly metastatic melanoma.

## Introduction

### Epidemiology of Melanoma

Melanoma is a type of malignant tumor derived from melanocytes in the basal layer of the epidermis. In the past few decades, the incidence of melanoma has increased rapidly in the developed countries, including the United States, Australia, and Spain, with higher incidence rates in fair-skinned individuals and older men (1). According to the latest SEER data, there were an estimated 106,000 new cases of melanoma in the United States in 2021, accounting for 5.6% of all cancer diagnoses, excluding non-melanoma skin cancer, which has become the fifth most commonly diagnosed cancer in the United States (2). The incidence of melanoma in Australia peaked around 2005, and continued decreases are expected owing to improvements in public health campaigns and the use of sunscreen (3).

In terms of prognosis, melanoma accounts for more than 80% of skin cancer-related deaths, despite representing a low percentage of total skin cancer cases (2). In the United States, with improvements in prevention, screening, diagnosis, and treatment (particularly targeted therapies and immunotherapies) in recent years, the 5-year overall survival rate of patients with melanoma has increased to more than 93%. Although the 5-year survival rate of patients with stage I–II disease is 99.4%, those of patients with stage III and IV disease are 68.0% and 29.8%, respectively (2).

### Subtypes of Melanoma

Melanoma can be divided into many clinical subtypes according to pathological type and molecular marker expression. Based on pathological type, melanoma can be divided into four common subtypes, as follows: superficial diffusion type, nodular type, malignant melanoma freckles melanoma, and acral freckle-like melanoma. Rarely, melanoma may also present as epithelioid type, which shows characteristics of fiber proliferation, as well as malignant pigmented nevus, balloon sample cells, spindle cells, and giant pigmented nevus malignant melanoma. The superficial diffuse type is most common in Caucasians, and acral freckle-like melanoma is most common in individuals of Asian and African descent (2).

Many studies have evaluated the relationships between molecular biological characteristics, clinical histological characteristics, and gene variations in melanoma in recent years, and the results have shown that specific types of melanoma are related to specific gene variations; therefore, scholars have proposed a new classification method based on molecular biological characteristics, which is more conducive to the application of clinical diagnosis and treatment (4–6). The new classification method can be divided into four basic types: extremum, mucous, chronic sun damage (CSD), and non-CSD (including unknown primary lesions). Notably, 28% of patients with sun damage-related melanoma harbor *KIT* gene mutations, whereas 10% harbor *BRAF* mutations and 5% harbor *NRAS* mutations. *KIT* gene mutations are more common in patients with acral and mucosal types, followed by *BRAF* mutations. The majority of non-CSD types, including trunk melanoma, exhibit *BRAF* gene V600E mutations (60%) or *NRAS* mutations (20%).

### Diagnosis of Melanoma

Similar to other diseases, typical clinical manifestations, physical examinations, imaging, and laboratory examinations (e.g., lactate dehydrogenase measurement) are commonly used for the diagnosis of melanoma (7, 8). The gold standard for melanoma diagnosis is pathological examination including immunohistochemical detection, which is of great value for evaluating melanoma stage, treatment, and prognosis (6). Immunohistochemistry is mainly used to assist in the identification of melanoma in pathological examination; for example, S-100, HMB-45, and vimentin are sensitive indicators for the specific diagnosis of melanoma (9, 10).

### Treatment of Melanoma

**Figure 1:**
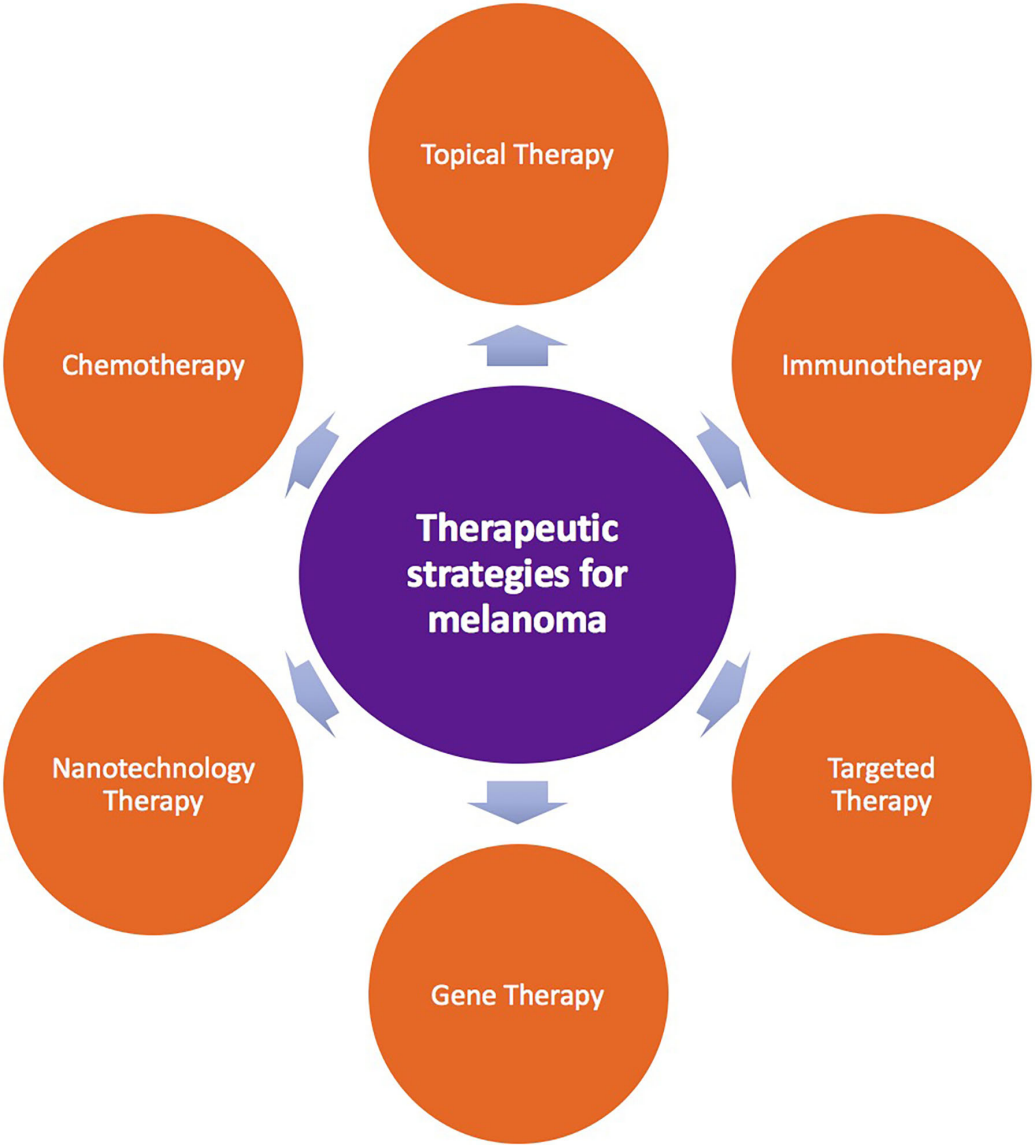
Therapeutic strategies for melanoma.

Current treatment methods for melanoma mainly include surgical treatment, adjuvant therapy, radiotherapy, photodynamic therapy, systemic therapy, and transfer to mucosal melanoma treatment (6, 11) (**Figure 1**). Common surgical treatments are biopsy, enlarged resection, sentinel lymph node biopsy, lymph node dissection, and in-transit metastasis (for patients with stage III disease) (6). Adjuvant therapy for melanoma is mainly based on the clinical stage and risk grade of patients (12). At present, although there is a broad consensus on appropriate adjuvant therapy for low-, medium-, and high-risk patients, there is still controversy regarding adjuvant therapy for very high-risk patients. Specific types of melanoma should be treated differently and individually. For example, adjuvant therapy with interferon treatment is important for high-risk patients. Radiotherapy for melanoma can be divided into adjuvant radiotherapy and palliative radiotherapy. Moreover, in patients with advanced melanoma, which is associated with a poor prognosis and a lack of effective treatments, systemic treatment based on internal medicine is typically applied. In recent years, breakthroughs in individualized targeted therapies and immunotherapies have led to improved outcomes in patients with advanced melanoma (13–16).

Because most targeted drugs are not widely used in the clinical setting in some countries, chemotherapy drugs, such as dacarbazine, temozolomide, paclitaxel, and carboplatin, are essential (17–22). Combined treatments have also been developed for mucosal melanomas originating from the mucous membranes of the head and neck, digestive tract, and genitourinary tract.

## Nanotechnology

Great progress has been made in the field of nanotechnology in recent decades, particularly with regard to the application of nanotechnology in medicine (23, 24). Nano-agents provide novel strategies for the treatment of many diseases owing to their unique characteristics of improving drug delivery. In the treatment of cancer, for example, conventional chemotherapy drugs do not specifically target the tumor and can also affect the body’s normal cells, resulting in various complications and seriously affecting patient’s quality of life. By contrast, nanotechnology can be used to achieve targeted drug delivery, improve pharmacokinetics and bioavailability, and overcome these barriers (25–28). Several different types of nano-agents have been used in clinical studies, including drug delivery, vaccine development, immunotherapy, and imaging diagnosis. However, the full potential of nanotechnology in clinical disease applications is far from being realized (29–31).

The most important approach involving nanomaterials is nanocarrier drug delivery systems (DDSs), which can transport active molecules, such as drugs, to the corresponding target in the body using nanoparticles as carriers. Compared with free drugs, DDSs are more specific and can greatly improve the therapeutic effect of drugs, while reducing potential side effects (32). The application of nano-DDSs in cancer treatment mainly involves using nanotechnology and materials to deliver drugs to tumor cells by passive or active targeting, thereby improving the therapeutic effects of drugs. Passive targeting typically involves the enhanced permeability and retention effect, whereas active targeting applies specific recognition and targeting of tumor-associated antigens by coupling monoclonal antibodies and peptides on the outer surface of DDSs (33–35).

### Nanotechnology and Melanoma

Melanoma is aggressive and easily metastatic; therefore, survival rates are low. The clinical treatment of melanoma includes a variety of treatment methods, such as drugs, surgery, radiotherapy. To improve the efficacy of drugs, various new multitarget drugs are often used in combination in the clinical setting. Nanotechnology-based DDSs, such as nanoliposomes, can play key roles in the clinical treatment of advanced melanoma because nanomaterials can target drug delivery at the cellular level by overcoming biological barriers in the body (32, 36, 37).

Nanomaterials have been used as DDSs for several types of cancer, and nanotechnology-based diagnosis and treatment of melanoma have also been proposed and investigated. First, as described above, because of the size and surface characteristics of nanomaterials, targeted drugs wrapped or loaded with nanomaterials easily cross the biological barrier and can be delivered specifically to melanoma cells, where they can exert their cytotoxic effects. Second, nanomaterials can reduce the side effects of off-target tissue toxicity and improve the efficacy of drugs. In addition, nanosystems may prevent the biodegradation of loaded drugs by the body, reduce drug removal, and prolong the half-life of the drug, allowing for dose reductions (38).

Various nanosystems, including lipid systems, inorganic nanoparticles, polymeric systems, and natural nanosystems, have been used for the diagnosis and treatment of melanoma (37, 39). For example, liposomes, solid lipid nanoparticles, and nanoemulsions have been developed as lipid nanosystems, whereas silica nanoparticles, gold nanoparticles, copper nanoparticles, and nanotubes have been used as common inorganic nanoparticle systems. Polymerization systems include polymeric micelles, nanospheres, polymeric nanoparticles, hydrogels, and dendritic macromolecules, and exosomes are a type of natural nanosystem (36).

#### Liposome Systems

Among various nanoparticle platforms, lipid systems deliver excellent performance in terms of physical stability, controlled release, and biocompatibility. Moreover, such lipid systems are usually biodegradable, show low side effects, and have relatively high physical stability. Therefore, lipid systems, including liposomes, solid lipid nanoparticles, and nanoemulsions, have been extensively studied and applied to clinical diseases.

Liposomes have been used as a type of double nano DDS in cancer treatment owing to their good pharmacokinetic characteristics. Furthermore, liposomes can significantly increase the circulation half-life of drugs and have been shown to enhance the efficacy of drugs in melanoma, particularly for drugs targeting the cell cycle, such as paclitaxel (40, 41). Bedikian et al. reported that the sheath package corpuscle (composed of sphingomyelin/cholesterol) of vincristine liposomes increased circulating half-life, accumulated at the tumor site, and increased therapeutic efficacy, enabling improved outcomes without altering drug dosage (40). Additionally, Matsumoto et al. also showed that cationic liposomes containing the human interferon B (HuIFNb) gene (*IAB-1*) showed higher antitumor activity than the treatment of melanoma with HuIFNb protein (42).

In addition, liposomes may have applications in the development of vaccines to treat and prevent melanoma. Gargett et al. studied a multicomponent dendritic cell-targeted vaccine, Lipovaxin-MM, which is administered intravenously for the treatment of metastatic melanoma (43). During the 12-week study period, Lipovaxin-MM was confirmed to be well tolerated without obvious immunogenicity and clinical toxicity, and the preliminary results suggested that Lipovaxin-MM may have applications as an immunotherapy in melanoma. Cancer vaccines, which have been studied extensively in basic and clinical trials, are characterized by the use of subunit antigens, which have relatively simple chemical compositions, manufacturing processes, and storage requirements; however, the tumor microenvironment is complex, particularly in advanced tumor models, and additional strategies may be required to achieve curative responses (44–46).

#### Inorganic Nanoparticles

Inorganic nanoparticles, including silica nanoparticles, gold nanoparticles, copper nanoparticles, and nanotubes, have good biocompatibility and enable simultaneous imaging and drug delivery (47, 48). However, these nanoparticles may not permit specific targeting to the affected region and generally need to be coupled with other targeting ligands. As an example, titanium dioxide is a weak dark compatible nanocrystal material with photocatalytic activity. The photoactivity of neat TiO_2_ is limited to the ultraviolet region, which limits its application in photodynamic therapy. Kozinska et al. applied functionalized fullerenes and surface-modified TiO_2_ as a photosensitizer for melanoma treatment and demonstrated that novel inorganic nanoparticles can achieve photodynamic killing of melanoma cells; this novel inorganic TiO_2_ nanoparticle complex was shown to have a longer retention time *in vivo* and to be nontoxic and stable under conditions without light irradiation (47). Ferreira et al. also evaluated a mouse melanoma model in which europium (III)-yttrium vanadate nanoparticles were functionalized with 3‐chloropropyl-trimethoxysilane with folic acid; compared with cisplatin alone, cisplatin nanoparticles modified with or without folic acid exhibited strong antitumor effects (49). Sapino et al. also constructed an inorganic nanoparticle system composed of aminopropyl-functionalized mesoporous silica nanoparticles (NH2-MSNs) as a topical carrier system for quercetin delivery and studied the effects of the topical carrier system on the proliferation of JR8 human melanoma cells; these NH2-MSNs were found to have strong antiproliferative effects in melanoma cells (50).

In addition, through advances in coordination chemistry, the abundant silanol groups (−Si−O−) on the surface of silica or in mesoporous channels have been directly used for radiolabeling of nonchelating compounds and easily modified with appropriate chelating compounds for chelate-based labeling. SiO_2_-based nano-inorganic material systems, including dense silica (dSiO_2_), mesoporous silica (MSN), biodegradable MSN (bMSN), and hollow MSN nanoparticles, have also been used in positron emission tomography imaging systems for patients with metastatic melanoma, providing a highly sensitive, noninvasive, and quantitative readout of organ/tissue distribution, pharmacokinetics, and tumor targeting efficiency. Thus, SiO_2_-based inorganic nanomaterials may have promising applications in the diagnosis of melanoma (51).

#### Polymeric Systems

Polymeric systems include polymeric micelles and nanospheres, polymeric nanoparticles, hydrogels, and dendrimers. Zou et al. provided a unique and secure platform for theranostic aggregates to construct a co-self-assembly of poly(ethylene glycol)-b-poly(dithiolane trimethylene carbonate-co-iodinated trimethylene carbonate) (PEG-P[DTC-IC]) and cRGD-PEG-P(DTC-IC) block copolymers as intelligent polymer antioxidants. Compared with that of iodinated nanosystems, the synthesis process of these polymeric nanosystems is simpler and overcomes the limitations of high viscosity and few applications. Similar to liposome systems, polymeric nanosystems can not only significantly enhance the computed tomography imaging of tumors but also mediate effective targeted chemotherapy for melanoma (52). Wang et al. constructed a cRGD-targeted polymeric oncolytic peptide LTX-315 and CpG adjuvant to combine with an anti-programmed cell death-1 (PD-1) antibody system; this approach provided strong, long-term immunotherapy for mouse malignant B16F10 melanoma and established a novel class of durable immunotherapy for hard-to-target and metastatic tumors, including melanoma (53). Although polymeric systems are diverse and have excellent nanodrug delivery properties, like other nanoplatforms, some polymeric nanoparticle systems exhibit poor physical stability and high toxicity, which limits their translation to the clinical setting.

#### Natural Nanosystems

Exosomes are nanovesicles containing various biomolecules. Exosomes are produced by cells through exocytosis and are taken up by target cells. They are involved in physiological and pathological processes and can transmit biological signals between local and distant cells. Therefore, exosomes can be modified as drug carriers for therapeutic intervention in diseases.

Exosomes can be used as natural nano DDSs owing to their unique characteristics (54–57). Exosomes are cellular vesicles composed of double membranes and have diameters ranging from 30 to 150 nm. Additionally, these vesicles can carry various biomolecules, including proteins, lipids, and nucleic acids, can pass through cell membranes and the blood–brain barrier, and can target specific cells (58–60). Circulating exosomes can be detected in blood samples, providing a promising diagnostic strategy for melanoma. In addition, exosomes have been evaluated as vehicles for delivery of therapeutic vaccines in melanoma (61). Monitoring exosomal programmed death ligand-1 (PD-L1) levels may enable prediction of the response to immunotherapy (60, 61). Upregulation of PD-L1 enhances interactions with the PD-1 receptor on T cells and triggers an immune checkpoint reaction, thereby allowing escape of immune monitoring (62, 63). However, in metastatic melanoma, melanoma cells typically express PD-L1, and the addition of interferon-γ stimulation increases exosomal PD-L1 levels; this results in the inhibition of CD8 T-cell function and promotes tumor growth, which explains the resistance to PD-1 and treatment failure observed in many patients. Accordingly, tumor PD-L1 has been used as a predictive biomarker of clinical response (64).

## Conclusions

Current therapy for melanoma has reached a limit of clinical responses. Nanotechnology has enabled the development of smaller, safer, and more accurate DDSs, and an increasing number of melanoma drugs are being packaged into nanocapsules as novel treatment systems. Some conventional melanoma drugs have low solubility in water buffer systems, poor bioavailability, rapid metabolism, and low stability, limiting their clinical potential and therapeutic use. Compared with traditional treatment methods, nano-encapsulation of drugs into the body can result in increased solubility, enhanced drug stability, improved epithelial permeability and bioavailability, longer half-life, increased tumor targeting, and minimal side effects. Thus, nano DDSs are expected to improve the therapeutic efficacy of the delivered drugs in patients with melanoma.

Future development challenges mainly focus on further understanding of the mechanisms that make nanosystems more effective than traditional drug formulations for melanoma. Overall, nano DDSs show good histocompatibility, enhanced drug targeting, low toxicity, and many other excellent characteristics, conferring broad applications in the diagnosis and treatment of melanoma, particularly metastatic melanoma.

## Author Contributions

All authors contributed to the design of the study and writing of the manuscript. KH, HZ, and JL undertook the research. YW, HZ, and NZ wrote the main manuscript text and prepared figures. NZ and HC revised the article critically for important intellectual content and final approval of the version to be submitted. All authors reviewed the manuscript.

## Conflict of Interest

The authors declare that the research was conducted in the absence of any commercial or financial relationships that could be construed as a potential conflict of interest.

## Publisher’s Note

All claims expressed in this article are solely those of the authors and do not necessarily represent those of their affiliated organizations, or those of the publisher, the editors and the reviewers. Any product that may be evaluated in this article, or claim that may be made by its manufacturer, is not guaranteed or endorsed by the publisher.

## Abbreviations

DDSs, drug delivery systems; HuIFNb, human interferon B; −Si−O−, silanol; dSiO_2_, dense silica; MSN, mesoporous silica; BMSN, biodegradable mesoporous silica; PD-1, programmed cell death-1.

## References

[1] Sung H Ferlay J Siegel RL Laversanne M Soerjomataram I Jemal A. Global Cancer Statistics 2020: GLOBOCAN Estimates of Incidence and Mortality Worldwide for 36 Cancers in 185 Countries. CA Cancer J Clin (2021) 71(3):209–49. doi: 10.3322/caac.21660 33538338

[2] Saginala K Barsouk A Aluru JS Rawla P Barsouk A. Epidemiology of Melanoma. Med Sci (Basel) (2021) 9(4):63. doi: 10.3390/medsci9040063 34698235 PMC8544364

[3] Whiteman DC Green AC Olsen CM. The Growing Burden of Invasive Melanoma: Projections of Incidence Rates and Numbers of New Cases in Six Susceptible Populations Through 2031. J Invest Dermatol (2016) 136(6):1161–71. doi: 10.1016/j.jid.2016.01.035 26902923

[4] Rabbie R Ferguson P Molina-Aguilar C Adams DJ Robles-Espinoza CD. Melanoma Subtypes: Genomic Profiles, Prognostic Molecular Markers and Therapeutic Possibilities. J Pathol (2019) 247(5):539–51. doi: 10.1002/path.5213 PMC6492003 30511391

[5] Swetter SM Tsao H Bichakjian CK Curiel-Lewandrowski C Elder DE Gershenwald JE. Guidelines of Care for the Management of Primary Cutaneous Melanoma. J Am Acad Dermatol (2019) 80(1):208–50. doi: 10.1016/j.jaad.2018.08.055 30392755

[6] Seth R Messersmith H Kaur V Kirkwood JM Kudchadkar R McQuade JL. Systemic Therapy for Melanoma: ASCO Guideline. J Clin Oncol (2020) 38(33):3947–70. doi: 10.1200/JCO.20.00198 32228358

[7] Garbe C Peris K Hauschild A Saiag P Middleton M Bastholt L. Diagnosis and Treatment of Melanoma. European Consensus-Based Interdisciplinary Guideline - Update 2016. Eur J Cancer (2016) 63:201–17. doi: 10.1016/j.ejca.2016.05.005 27367293

[8] Nenclares P Ap Dafydd D Bagwan I Begg D Kerawala C King E. Head and Neck Mucosal Melanoma: The United Kingdom National Guidelines. Eur J Cancer (2020) 138:11–8. doi: 10.1016/j.ejca.2020.07.017 32829104

[9] Botella-Estrada R Boada-Garcia A Carrera-Alvarez C Fernandez-Figueras M Gonzalez-Cao M Moreno-Ramirez D. Clinical Practice Guideline on Melanoma From the Spanish Academy of Dermatology and Venereology (AEDV). Actas Dermosifiliogr (Engl Ed) (2021) 112(2):142–52. doi: 10.1016/j.ad.2020.07.003 32721390

[10] Katz I O’Brien B Clark S Thompson CT Schapiro B Azzi A. Assessment of a Diagnostic Classification System for Management of Lesions to Exclude Melanoma. JAMA Netw Open (2021) 4(12):e2134614. doi: 10.1001/jamanetworkopen.2021.34614 34889949 PMC8665368

[11] Aziz HA Gastman BR Singh AD. Management of Conjunctival Melanoma: Critical Assessment of Sentinel Lymph Node Biopsy. Ocul Oncol Pathol (2015) 1(4):266–73. doi: 10.1159/000381719 PMC4847666 27171676

[12] Wada-Ohno M Ito T Furue M. Adjuvant Therapy for Melanoma. Curr Treat Options Oncol (2019) 20(8):63. doi: 10.1007/s11864-019-0666-x 31236710

[13] Albittar AA Alhalabi O Glitza Oliva IC. Immunotherapy for Melanoma. Adv Exp Med Biol (2020) 1244:51–68. doi: 10.1007/978-3-030-41008-7_3 32301010

[14] Onitilo AA Wittig JA. Principles of Immunotherapy in Melanoma. Surg Clin North Am (2020) 100(1):161–73. doi: 10.1016/j.suc.2019.09.009 31753110

[15] Klebaner D Saddawi-Konefka R Finegersh A Yan CH Califano JA 3rd London NR. Immunotherapy in Sinonasal Melanoma: Treatment Patterns and Outcomes Compared to Cutaneous Melanoma. Int Forum Allergy Rhinol (2020) 10(9):1087–95. doi: 10.1002/alr.22628 32623838

[16] Robert C Long GV Brady B Dutriaux C Maio M Mortier L. Nivolumab in Previously Untreated Melanoma Without BRAF Mutation. N Engl J Med (2015) 372(4):320–30. doi: 10.1056/NEJMoa1412082 25399552

[17] Chapman PB Hauschild A Robert C Haanen JB Ascierto P Larkin J. Improved Survival With Vemurafenib in Melanoma With BRAF V600E Mutation. N Engl J Med (2011) 364(26):2507–16. doi: 10.1056/NEJMoa1103782 PMC3549296 21639808

[18] Ott PA Chang J Madden K Kannan R Muren C Escano C. Oblimersen in Combination With Temozolomide and Albumin-Bound Paclitaxel in Patients With Advanced Melanoma: A Phase I Trial. Cancer Chemother Pharmacol (2013) 71(1):183–91. doi: 10.1007/s00280-012-1995-7 23064957

[19] Middleton MR Grob JJ Aaronson N Fierlbeck G Tilgen W Seiter S. Randomized Phase III Study of Temozolomide Versus Dacarbazine in the Treatment of Patients With Advanced Metastatic Malignant Melanoma. J Clin Oncol (2000) 18(1):158–66. doi: 10.1200/JCO.2000.18.1.158 10623706

[20] Larkin J Minor D D’Angelo S Neyns B Smylie M Miller WH Jr.. Overall Survival in Patients With Advanced Melanoma Who Received Nivolumab Versus Investigator’s Choice Chemotherapy in CheckMate 037: A Randomized, Controlled, Open-Label Phase III Trial. J Clin Oncol (2018) 36(4):383–90. doi: 10.1200/JCO.2016.71.8023 PMC6804912 28671856

[21] Kato K Cho BC Takahashi M Okada M Lin CY Chin K. Nivolumab Versus Chemotherapy in Patients With Advanced Oesophageal Squamous Cell Carcinoma Refractory or Intolerant to Previous Chemotherapy (ATTRACTION-3): A Multicentre, Randomised, Open-Label, Phase 3 Trial. Lancet Oncol (2019) 20(11):1506–17. doi: 10.1016/S1470-2045(19)30626-6 31582355

[22] Takahashi M Kato K Okada M Chin K Kadowaki S Hamamoto Y. Nivolumab Versus Chemotherapy in Japanese Patients With Advanced Esophageal Squamous Cell Carcinoma: A Subgroup Analysis of a Multicenter, Randomized, Open-Label, Phase 3 Trial (ATTRACTION-3). Esophagus (2021) 18(1):90–9. doi: 10.1007/s10388-020-00794-x PMC7794205 33170461

[23] Gharpure KM Wu SY Li C Lopez-Berestein G Sood AK. Nanotechnology: Future of Oncotherapy. Clin Cancer Res (2015) 21(14):3121–30. doi: 10.1158/1078-0432.CCR-14-1189 PMC4505622 26180057

[24] Gao D Guo X Zhang X Chen S Wang Y Chen T. Multifunctional Phototheranostic Nanomedicine for Cancer Imaging and Treatment. Mater Today Bio (2020) 5:100035. doi: 10.1016/j.mtbio.2019.100035 PMC7083767 32211603

[25] Goldberg MS. Improving Cancer Immunotherapy Through Nanotechnology. Nat Rev Cancer (2019) 19(10):587–602. doi: 10.1038/s41568-019-0186-9 31492927

[26] Xu C Hong H Lee Y Park KS Sun M Wang T. Efficient Lymph Node-Targeted Delivery of Personalized Cancer Vaccines With Reactive Oxygen Species-Inducing Reduced Graphene Oxide Nanosheets. ACS Nano (2020) 14(10):13268–78. doi: 10.1021/acsnano.0c05062 PMC7606610 32902245

[27] Yadav N Dahiya T Chhillar AK Rana JS Mohan H. Promising Applications of Nanotechnology in Cancer Diagnostics and Therapeutics. Curr Pharm Biotechnol (2021). doi: 10.2174/1389201023666211222165508 34951360

[28] Zhu D Li Y Zhang Z Xue Z Hua Z Luo X. Recent Advances of Nanotechnology-Based Tumor Vessel-Targeting Strategies. J Nanobiotechnol (2021) 19(1):435. doi: 10.1186/s12951-021-01190-y PMC8686559 34930293

[29] Zhou J Krishnan N Jiang Y Fang RH Zhang L. Nanotechnology for Virus Treatment. Nano Today (2021) 36:101031. doi: 10.1016/j.nantod.2020.101031 33519948 PMC7836394

[30] Sahle FF Kim S Niloy KK Tahia F Fili CV Cooper E. Nanotechnology in Regenerative Ophthalmology. Adv Drug Delivery Rev (2019) 148:290–307. doi: 10.1016/j.addr.2019.10.006 PMC7474549 31707052

[31] Kirtane AR Verma M Karandikar P Furin J Langer R Traverso G. Nanotechnology Approaches for Global Infectious Diseases. Nat Nanotechnol (2021) 16(4):369–84. doi: 10.1038/s41565-021-00866-8 33753915

[32] Bensa V Calarco E Giusto E Perri P Corrias MV Ponzoni M. Retinoids Delivery Systems in Cancer: Liposomal Fenretinide for Neuroectodermal-Derived Tumors. Pharm (Basel) (2021) 14(9):854. doi: 10.3390/ph14090854 PMC8466194 34577553

[33] Torchilin V. Tumor Delivery of Macromolecular Drugs Based on the EPR Effect. Adv Drug Delivery Rev (2011) 63(3):131–5. doi: 10.1016/j.addr.2010.03.011 20304019

[34] Pastorino F Brignole C Di Paolo D Perri P Curnis F Corti A. Overcoming Biological Barriers in Neuroblastoma Therapy: The Vascular Targeting Approach With Liposomal Drug Nanocarriers. Small (2019) 15(10):e1804591. doi: 10.1002/smll.201804591 30706636

[35] Attia MF Anton N Wallyn J Omran Z Vandamme TF. An Overview of Active and Passive Targeting Strategies to Improve the Nanocarriers Efficiency to Tumour Sites. J Pharm Pharmacol (2019) 71(8):1185–98. doi: 10.1111/jphp.13098 31049986

[36] Battaglia L Scomparin A Dianzani C Milla P Muntoni E Arpicco S. Nanotechnology Addressing Cutaneous Melanoma: The Italian Landscape. Pharmaceutics (2021) 13(10):1617. doi: 10.3390/pharmaceutics13101617 34683910 PMC8540596

[37] Mishra H Mishra PK Ekielski A Jaggi M Iqbal Z Talegaonkar S. Melanoma Treatment: From Conventional to Nanotechnology. J Cancer Res Clin Oncol (2018) 144(12):2283–302. doi: 10.1007/s00432-018-2726-1 PMC11813321 30094536

[38] Gmeiner WH Ghosh S. Nanotechnology for Cancer Treatment. Nanotechnol Rev (2015) 3(2):111–22. doi: 10.1515/ntrev-2013-0013 PMC4465796 26082884

[39] Kurakula M Chen L Tiwari AK Srinivas NR Dash RP Panizzi PR. Recent Advances in Lipid-Based Nanovesicular Delivery Systems for Melanoma Therapy. Crit Rev Ther Drug Carrier Syst (2021) 38(4):1–38. doi: 10.1615/CritRevTherDrugCarrierSyst.2021034903 34369738

[40] Bedikian AY Vardeleon A Smith T Campbell S Namdari R. Pharmacokinetics and Urinary Excretion of Vincristine Sulfate Liposomes Injection in Metastatic Melanoma Patients. J Clin Pharmacol (2006) 46(7):727–37. doi: 10.1177/0091270006288953 16809798

[41] Saengkrit N Saesoo S Srinuanchai W Phunpee S Ruktanonchai UR. Influence of Curcumin-Loaded Cationic Liposome on Anticancer Activity for Cervical Cancer Therapy. Colloids Surf B Biointerf (2014) 114:349–56. doi: 10.1016/j.colsurfb.2013.10.005 24246195

[42] Matsumoto K Kubo H Murata H Uhara H Takata M Shibata S. A Pilot Study of Human Interferon Beta Gene Therapy for Patients With Advanced Melanoma by *In Vivo* Transduction Using Cationic Liposomes. Jpn J Clin Oncol (2008) 38(12):849–56. doi: 10.1093/jjco/hyn114 18945721

[43] Gargett T Abbas MN Rolan P Price JD Gosling KM Ferrante A. Phase I Trial of Lipovaxin-MM, a Novel Dendritic Cell-Targeted Liposomal Vaccine for Malignant Melanoma. Cancer Immunol Immunother (2018) 67(9):1461–72. doi: 10.1007/s00262-018-2207-z PMC11028356 30014244

[44] Lu L Yan H Shyam-Sundar V Janowitz T. Cross-Sectional and Longitudinal Analysis of Cancer Vaccination Trials Registered on the US Clinical Trials Database Demonstrates Paucity of Immunological Trial Endpoints and Decline in Registration Since 2008. Drug Des Devel Ther (2014) 8:1539–53. doi: 10.2147/DDDT.S65963 PMC4189706 25302014

[45] Faries MB Morton DL. Therapeutic Vaccines for Melanoma: Current Status. BioDrugs (2005) 19(4):247–60. doi: 10.2165/00063030-200519040-00004 16128607

[46] Liu Q Das M Liu Y Huang L. Targeted Drug Delivery to Melanoma. Adv Drug Delivery Rev (2018) 127:208–21. doi: 10.1016/j.addr.2017.09.016 28939379

[47] Kozinska A Zadlo A Labuz P Broniec A Pabisz P Sarna T. The Ability of Functionalized Fullerenes and Surface-Modified TiO2 Nanoparticles to Photosensitize Peroxidation of Lipids in Selected Model Systems. Photochem Photobiol (2019) 95(1):227–36. doi: 10.1111/php.13053 30466182

[48] Wang J Sui L Huang J Miao L Nie Y Wang K. MoS2-Based Nanocomposites for Cancer Diagnosis and Therapy. Bioact Mater (2021) 6(11):4209–42. doi: 10.1016/j.bioactmat.2021.04.021 PMC8102209 33997503

[49] Ferreira NH Furtado RA Ribeiro AB de Oliveira PF Ozelin SD de Souza LDR. Europium(III)-Doped Yttrium Vanadate Nanoparticles Reduce the Toxicity of Cisplatin. J Inorg Biochem (2018) 182:9–17. doi: 10.1016/j.jinorgbio.2018.01.014 29407869

[50] Sapino S Ugazio E Gastaldi L Miletto I Berlier G Zonari D. Mesoporous Silica as Topical Nanocarriers for Quercetin: Characterization and *In Vitro* Studies. Eur J Pharm Biopharm (2015) 89:116–25. doi: 10.1016/j.ejpb.2014.11.022 25478737

[51] Ni D Jiang D Ehlerding EB Huang P Cai W. Radiolabeling Silica-Based Nanoparticles *via* Coordination Chemistry: Basic Principles, Strategies, and Applications. Acc Chem Res (2018) 51(3):778–88. doi: 10.1021/acs.accounts.7b00635 PMC5878690 29489335

[52] Zou Y Wei Y Sun Y Bao J Yao F Li Z. Cyclic RGD-Functionalized and Disulfide-Crosslinked Iodine-Rich Polymersomes as a Robust and Smart Theranostic Agent for Targeted CT Imaging and Chemotherapy of Tumor. Theranostics (2019) 9(26):8061–72. doi: 10.7150/thno.37184 PMC6857068 31754381

[53] Wang M Geilich BM Keidar M Webster TJ. Killing Malignant Melanoma Cells With Protoporphyrin IX-Loaded Polymersome-Mediated Photodynamic Therapy and Cold Atmospheric Plasma. Int J Nanomed (2017) 12:4117–27. doi: 10.2147/IJN.S129266 PMC5459981 28615940

[54] Kalluri R LeBleu VS. The Biology, Function, and Biomedical Applications of Exosomes. Science (2020) 367(6478):eaau6977. doi: 10.1126/science.aau6977 32029601 PMC7717626

[55] Sun Z Shi K Yang S Liu J Zhou Q Wang G. Effect of Exosomal miRNA on Cancer Biology and Clinical Applications. Mol Cancer (2018) 17(1):147. doi: 10.1186/s12943-018-0897-7 30309355 PMC6182840

[56] Raposo G Stoorvogel W. Extracellular Vesicles: Exosomes, Microvesicles, and Friends. J Cell Biol (2013) 200(4):373–83. doi: 10.1083/jcb.201211138 PMC3575529 23420871

[57] Buratta S Tancini B Sagini K Delo F Chiaradia E Urbanelli L. Lysosomal Exocytosis, Exosome Release and Secretory Autophagy: The Autophagic- and Endo-Lysosomal Systems Go Extracellular. Int J Mol Sci (2020) 21(7):2576. doi: 10.3390/ijms21072576 PMC7178086 32276321

[58] Yang F Ning Z Ma L Liu W Shao C Shu Y. Exosomal miRNAs and miRNA Dysregulation in Cancer-Associated Fibroblasts. Mol Cancer (2017) 16(1):148. doi: 10.1186/s12943-017-0718-4 28851377 PMC5576273

[59] Han Y Jia L Zheng Y Li W. Salivary Exosomes: Emerging Roles in Systemic Disease. Int J Biol Sci (2018) 14(6):633–43. doi: 10.7150/ijbs.25018 PMC6001649 29904278

[60] Chen G Huang AC Zhang W Zhang G Wu M Xu W. Exosomal PD-L1 Contributes to Immunosuppression and is Associated With Anti-PD-1 Response. Nature (2018) 560(7718):382–6. doi: 10.1038/s41586-018-0392-8 PMC6095740 30089911

[61] He C Zheng S Luo Y Wang B. Exosome Theranostics: Biology and Translational Medicine. Theranostics (2018) 8(1):237–55. doi: 10.7150/thno.21945 PMC5743472 29290805

[62] Chen L Han X. Anti-PD-1/PD-L1 Therapy of Human Cancer: Past, Present, and Future. J Clin Invest (2015) 125(9):3384–91. doi: 10.1172/JCI80011 PMC4588282 26325035

[63] Topalian SL Taube JM Anders RA Pardoll DM. Mechanism-Driven Biomarkers to Guide Immune Checkpoint Blockade in Cancer Therapy. Nat Rev Cancer (2016) 16(5):275–87. doi: 10.1038/nrc.2016.36 PMC5381938 27079802

[64] Ribas A Hamid O Daud A Hodi FS Wolchok JD Kefford R. Association of Pembrolizumab With Tumor Response and Survival Among Patients With Advanced Melanoma. JAMA (2016) 315(15):1600–9. doi: 10.1001/jama.2016.4059 27092830

